# How a teaching rotation in medical school affects graduates’ subsequent careers

**DOI:** 10.1007/s40037-016-0302-4

**Published:** 2016-10-18

**Authors:** Anne T. Kloek, Angela C.M. van Zijl, Olle T.J. ten Cate

**Affiliations:** 1 Academic Medical Center, Amsterdam, The Netherlands; 2 University Medical Center, Utrecht, The Netherlands

**Keywords:** Teaching rotation, Educational career, Medical graduates

## Abstract

**Introduction:**

Teaching opportunities and teacher courses for medical students are increasingly offered by medical schools but little has been investigated about their long-term effect. The aim of our study was to investigate the long-term career effect of an intensive elective teaching experience for final year medical students.

**Methods:**

We approached UMC Utrecht medical graduates who had taken a final year, 6‑week full time student teaching rotation (STR) elective, 6 to 9 years after graduation, with an online survey to ask about their educational activities and obtained teaching certificates, their current roles related to education, and their appreciation of the rotation, even if this was a long time ago. In addition, we surveyed control groups of students who had not taken the STR, divided into those who had expressed interest in the STR but had not been placed and those who had not expressed such interest.

**Results:**

We received responses from 50 STR graduates and 88 non-STR graduates (11 with interest and 77 without interest in the STR). STR graduates were more educationally active, had obtained more university teaching certificates and were more enthusiastic teachers. However, we could not exclude confounding, caused by a general interest in education even before the STR.

**Conclusions:**

Our findings indicate a high appreciation of the student teaching rotation and a likely but not proven long-term association between STR participation and building an educational career.

## What this paper adds

- Teachings skills are increasingly acknowledged as important for medical graduates, but little is known about the long-term effects of training medical students in teaching skills. We performed a 6 to 9 year follow-up survey of medical graduates with a Student Teaching Rotation (STR) experience and medical graduates without STR experience. The graduates were asked about their current teaching activities, obtained teaching certificates, appreciation of teaching and teaching career building. We concluded that the STR is a valuable elective rotation for senior medical students and we recommend introducing this rotation in all medical curricula.

## Introduction

The continuum of medical education to independent practice is among the longest tracks in higher education, spanning over 10 to 15 years in most jurisdictions [1]. This significant investment in training the medical workforce is not paralleled by the training of their educators in the skills of teaching and coaching. Yet much of what medical students learn happens through teaching by residents and physicians. Conversely, most residents and practising physicians educate students. Education may be viewed as a logical professional obligation since Hippocratic times, indeed as an inherent characteristic of any profession. Teaching is often assumed to be a skill present in medical graduates but in most medical schools students are not trained to teach. Learning the act of teaching from role models is becoming less obvious as trainees and their supervisors have less time in clinical practice to observe each other [2, 3] and teaching in classroom settings is hardly ever observed for the purpose of acquiring teaching skills.

In the last three decades, scholarship in medical education has considerably increased, as evidenced by the existence over 30 medical education journals, an increasing number of Master’s and PhD training programmes in health professions education, many conferences and associations for medical education, and foremost a significant change in the structure and methods of many medical curricula in the Western world, both in preclinical and clinical education. Despite this increased attention for medical education, the majority of medical teachers are not formally trained to adapt their teaching. Continuing to teach as one was taught may have been justified in the past, at a time with only incremental development and change in curricula. Now, with new pedagogies, new technologies, changing clinical workplace settings and rapidly increasing medical knowledge, teachers can no longer rely on using the teaching methods they themselves were taught by.

Preparing clinicians to teach is predominantly practised as voluntary faculty development, as an add-on to patient care obligations and, in universities, to research practice – tasks that usually require formal training. A small but increasing number of medical schools, however, have now begun to offer teacher training to medical students, mostly as electives, incidentally as a mandatory course [4–6] and some schools offer longer elective teacher training pathways [7]. Training teaching skills requires practice opportunities. Teaching opportunities for students include near peer teaching programmes [8, 9] and outreach initiatives with students teaching in secondary and primary education [4, 10]. Students usually appreciate being taught by near peers [11, 12] and there are indications that learning effects may be as good as those from more experienced teachers [13–15], while teaching itself also fosters learning [16, 17]. Theoretical underpinnings for these effects can be found in the literature [11, 18]. Career trajectories for teachers in medicine may thus start as early as in undergraduate medical training.

University Medical Center Utrecht (UMCU) offers a six-week elective rotation in teaching skills to about 10 % of all final year medical students [19], now over 400 students in a 12-year period. Our impression is that current generations of medical graduates from the UMCU programme are much more interested in education than they were in the past. We were interested to investigate whether those students who take this elective teaching rotation more readily pursue a teaching career in medicine.

The aim of our study was to examine the long-term effect of participating in a student teaching rotation (STR) on the educational career of medical graduates. We compared STR graduates with two other groups: non-STR graduates without interest in a teaching rotation and non-STR graduates who had wanted to take part but were not selected because of limited capacity. This selection for admission was done by lot or administrative criteria, as there has always been more interest for the teaching rotation than places available. We wanted to examine whether the graduated teaching rotation trainees would be more frequently educationally active and more interested in teaching activities and a teaching career and whether the rotations had prepared them well for teaching activities or a teaching career.

## Methods

### Content of the UMCU student teaching rotation

The six-week STR is offered in eight periods throughout the year and is coordinated by the Center for Research and Development of Education at UMCU. During the teaching rotation, students are attached to an undergraduate curriculum course and they gain experience with a broad spectrum of teacher roles. They must teach junior medical students (occasionally nursing or other students) for a minimum of 30 hours. Next, they must study medical education literature and sit a quiz on 37 core topics, observe other teachers and provide structured feedback, complete a small educational development project to improve the course they are participating in, they must write a small literature review on a medical education topic of choice and must practise writing test items. The rotation students meet regularly with their colleague rotation students in supervised groups of three to seven, discuss a video recording of their own teaching and discuss educational matters with an educator-supervisor present.

Students who complete the STR receive a student teaching certificate which can be shown at job and residency applications. The capacity of the STR was on average 35 per year, but the interest among students has always been greater.

### Participants

In the spring of 2013 we directly approached all 221 graduates who had taken the STR and of whom a current email address could be retrieved and approached other graduates (approximately 600) through the newsletter of the Utrecht University Young Alumni Network; all had graduated in the period 2004–2007 and we assumed they had had time (up to nine years) to establish a career that could possibly include education. Written informed consent was obtained by email from all participants prior to study participation. The graduates were invited by email to take part in the survey, either directly or through the newsletter. After one week a reminder was sent to those graduates who had received a personal email request. An additional effort was made to reach STR graduates. If they did not respond to the personal survey invitation they were contacted by telephone.

### Materials and procedures

A 52-item questionnaire was designed including closed format and open-ended questions about medical and educational career options, future education ambitions and preparation and enthusiasm for educational activities. The closed format items asked for a yes/no response (e. g., Have you been involved with education after graduation?), or multiple options (e. g., Why does your employment include teaching: in the job description – added later by employer – requested by colleagues – initiated by oneself). Open question included facts (e. g., What is your age? What is your discipline? What are your estimated hours of teaching per week?) or opinions and explanations (e. g., Why did you or did you not pursue a faculty teaching certificate?) and open space for comments. The questionnaire was pretested with senior medical students. The questionnaire included items about age, gender, current discipline, current employment and role. Next, items included current educational activities in five domains derived from Harden & Crosby (instructional practice, assessment, educational management, individual mentoring and educational development) [20] and items on the period, amount and frequency of educational activities. Finally, respondents were asked whether they had actively sought educational tasks or if the tasks were just given to them, and whether they had obtained a faculty teaching certificate, how they valued their teaching activities and if they felt well prepared for teaching as a medical graduate.

### Data analysis

Central estimators and dispersion measures were explored across the three groups: teaching rotation graduates, graduates who had applied to the teaching rotation but had not been admitted, and graduates who had not expressed interest in the teaching rotation (STR graduates, non-STR graduates with interest and non-STR graduates without interest, respectively). Discrete variables were expressed as numbers with percentages and continuous variables with skewed data were expressed as median values and interquartile ranges. Differences between the three groups were tested by Kruskal-Wallis or chi-square for continuous variables with skewed data and frequencies, respectively. Differences between pairs of groups were tested with the Mann-Whitney *U *test or chi-square in case of medians and frequencies, respectively.

Respondents with missing data were not excluded from all analyses, but only from the analysis of the specific questions for which a value was missing. For example, if someone did not finish the questionnaire and answered questions about educational activity but no further questions about teaching enthusiasm, the answers about educational activity were used in the analysis.

Statistical significance was considered reached at *p* < 0.05 (two-sided). All statistical analyses were performed with SPSS version 20.0 for Windows (IBM, Armonk, New York, USA).

## Results

### Response rates

A total of 84 students had taken the STR in the years 2004–2007. A total of 151 medical graduates participated in the study. Of these, 94 had responded to the email invitation (94/22; 42.5 %) including 4 STR graduates who were added after contact by telephone, and 57 had responded to the newsletter appeal (57/600; 9.5 %). Fully completed questionnaires were received from 138 graduates.

Of the participants who completed the questionnaire, 50 (36 %) were STR graduates and 88 (64 %) non-STR graduates, including 11 who had wanted to take the STR but were not placed. We found no statistical differences between the three groups in baseline characteristics of age, gender, choice of specialty or role (resident physician, non-resident physician, PhD student or other).

### Educationally active

After graduation, there was a difference in educational activity between the three groups (Table 1). Non-STR graduates without interest were significantly less educationally active than non-STR graduates with interest and the STR graduates together (*χ* ^2^ = 4.247, *p* = 0.039, Cramer’s V: 0.198). We found no significant difference in educational activity between the non-STR graduates with interest and the STR graduates.

The number of years and hours per week spent on educational activities after graduation did not differ between the groups and the three groups did not vary in current educational activities as part of their work.

### University teaching certificates obtained

Of the STR graduates, 28 % had obtained a basic university teaching certificate and 13 % were on track to obtain one. STR graduates had more often obtained or were on track to obtain a basic university teaching certificate than non-STR graduates without STR interest (*χ* ^2^ = 20.6, *p* ≤ 0.001, Cramer’s V: 0.44).

STR graduates and the non-STR graduates with interest showed more own initiative to obtain a basic university teaching certificate than the non-STR graduates without interest (*χ* ^2^ = 6.7, *p* = 0.036, Cramer’s V = 0.527).

STR graduates were more often on track to obtain a senior teaching certificate than non-STR graduates with interest and non-STR graduates without interest (*χ* ^2^ = 4.8, *p* = 0.028, Cramer’s V = 0.240). At the time of the survey, no-one as yet had obtained the senior teaching certificate.

### Educational plans

The future perspectives on educational activities varied between the three groups. Particularly the STR graduates and non-STR graduates with interest were more inclined to participate in future educational activities than the non-STR graduates without interest, as could be expected (*χ* ^2^ = 5.4, *p* = 0.021, Cramer’s V:0.20). When asked what an ideal time distribution between patient care, research, education and other activities would be, we found that the STR graduates would want to spend most time on education, followed by the non-STR-with-interest group (*Kruskal-Wallis: χ* ^2^ *= 18.5, p < 0.001*).

### Initiative and appreciation of teaching

Educational activities could be part of the respondents’ job or added later, either on their own initiative, or requested by a supervisor or by someone else. The three groups showed no significant differences in taking initiative for educational activities. Also, differences in added educational tasks without extending a contract (i. e. voluntary extra teaching) were not significant between the three groups.

Most respondents appreciated educational activities. Only a few participants in the non-STR without interest group showed moderate appreciation, in contrast to the very positive judgment by the STR graduates (*χ* ^2^ = 6.407, *p* = 0.041, Cramer’s V = 0.247). The subjects were also asked how they felt prepared for education. Particularly non-STR-without-interest graduates felt less well prepared compared with STR graduates (*χ* ^2^ = 12.88, *p* = 0.005, Cramer’s V: 0.354).

### Evaluation of the student teaching rotation

The STR graduates were asked to rate the rotation in hindsight and estimate how this course contributed to the feeling of being prepared for education, and also whether it had affected their education career. The results show a positive evaluation of the teaching rotation (neutral (2 %, *n* = 2), positive (48 %, *n* = 24), very positive (44 %, *n* = 22), extremely positive (6 %, *n* = 3)). STR graduates felt well prepared for education based on their teaching experience (24 %, *n* = 10), the introduction to medical education within the rotation (19 %, *n* = 8) and training in teaching skills and theory of medical education (29 %, *n* = 12). One participant noted that the rotation did not add much to pre-existing teaching experience. The rotation had stimulated and confirmed the personal interest in education according to 64 % of the STR graduates (*n* = 26).

## Discussion

Training students in teaching skills during a dedicated rotation devoted to supervised teaching appears to benefit both the senior students trained and the junior students being taught [8, 14, 19]. The present study shows that graduates from a student teaching rotation remain more educationally active in terms of pursuing teaching certificates and their desire to spend job time on education, but we were surprised to see how non-STR graduates generally appreciated teaching too. STR graduates showed more enthusiasm about teaching and felt better prepared to educate than graduates who had not been interested in a student teaching rotation. STR graduates were more engaged with education and personal development in education by obtaining more advanced teaching certificates compared with non-STR graduates and also intended to spend more working hours on education.

The differences in career development of students graduating with and without a student teaching certificate were not as large as we had expected. We cannot draw firm conclusions about an effect of the rotations per se on career development, as the general interest in education is likely a confounding factor. General interest in education may have caused career-related education activities, rather that the rotation itself. A reason for this general interest could be that all students in this medical curriculum also receive a mandatory one-week teacher training course that is generally well received [5]. Moreover, the non-STR graduates with interest may have done similar courses or extracurricular activities to fulfil their interest in medical education.

The results of the present study align with our hypothesis that students taking part in a teaching rotation frequently remain active in teaching and are more enthusiastic teachers. Our results are consistent with observations of short-term effects of a teaching rotation reported by others. Smith et al. showed how a teaching rotation resulted in improvement in teaching and leadership; 57 % of the participants were engaged in medical education development projects 11 months later [21]. Andreatta et al. showed that after two years the participants of a 5-day teaching experience for medical students had incorporated the theory of medical education and the experience of the teaching rotation in their careers [22].

Our study has a number of important limitations. We had a low response rate to the newsletter appeal, although we did not expect a high response in advance. The expectations were low because the invitation was not personal and it is known that the newsletter is not read thoroughly. We cannot exclude that a specific group of alumni did respond to the newsletter invitation. We were only able to include a moderate sample of non-STR graduates who had been interested in a rotation but were not accepted. We estimate that group for the period chosen to be about 50. Also, the non-STR group without STR interest sample was small. The 50 STR and 88 non-STR graduate samples show quite a different representation of these respective populations, the second group being much more selective. Our conclusions should therefore be read with some caution. However, we do not expect that larger comparison groups would have contradicted our conclusions. On the contrary, it is not unlikely that non-STR respondents had a relatively high affinity with teaching to make them respond to our questionnaire. They could have contaminated the control group but we were not able to correct for this. These effects could have reduced existing differences between the groups, and mitigated our conclusions, but this, admittedly, is speculation.

The reason why we did not use a historical comparison group, i. e. a group that had graduated before the STR existed, is that the difference in historical time frame could have shown an effect caused by other developments than the STR. As we noted in the introduction, there has been a rapid increase in attention for education and faculty development, which would make such cohorts less comparable. On the other hand our current cohort of students as a whole may be more interested in education than previous generations, because of all teaching opportunities offered.

To the best of our knowledge, this is the first study reporting the long-term career effect of a teaching rotation or course longer than the week Andreatta et al. reported on [22]. Furthermore, the present study is based not only on opinions but also on more quantitative measures, such as estimated time spent on educational activities and includes a special control group, those participants who wanted to take part of the teaching rotation but did not obtain a place by lot.

## Conclusion

In sum, a student teaching rotation likely stimulates graduates in building an educational career and is therefore a valuable elective course in the medical curriculum. We recommend introducing elective teaching rotations in medical curricula for senior medical students.

**Table 1:** Educational activities, appreciation and plans of graduates with and without experience in a student teaching rotation

	STR graduates	Non-STR graduates with STR interest	Non-STR graduates without STR interest	Significant group difference	Missing cases per group 1/2/3
*Educationally active*	*n = 50*	*n = 11*	*n = 77*	*–*	*All respondents*
Since graduation	46 (94 %)	11 (100 %)	63 (82 %)	1 + 2 vs 3*	1/0/0
Number of years (IQR)	3.0 (3.3)	1.5 (3.0)	3.0 (4.5)	ns	5/0/4
Hours per week (IQR)	1.0 (5.0)	1.0 (2.0)	1.0 (2.0)	ns	3/0/4
Currently active	24 (50 %)	7 (64 %)	40 (55 %)	ns	2/0/4
BKO Obtained/in a track	19 (41 %)	2 (18 %)	4 (5 %)	1 vs 3, 1 vs 2 + 3, 1 + 2 vs 3, 1 vs 2 vs 3***	4/0/5
Own initiative to obtain BKO	15/19 (83 %)	2/2 (100 %)	1/4 (25 %)	1 vs 2 vs 3*	None
SKO Obtained/in a track	4 (9 %)	0(0 %)	0(0 %)	1 vs 3, 1vs 2 + 3, 1 vs 2 vs 3*	4/0/5
Own initiative to obtain SKO	2/3 (67 %)	–	–	ns	1/–/–
Plans to remain active	40 (85 %)	11 (100 %)	52 (71 %)	1 vs 2 vs 3, 1 + 2 vs 3*	3/0/4
Preferred of job time spent on education in percentages (IQR)	20 (10)	10 (10)	10 (10)	1 + 2 vs 3**, 1 vs 3, 1vs 2 + 3, 1 vs 2 vs 3***	3/0/4
*Enthusiasm about teaching*	*n = 24*	*n=7*	*n = 40*	*–*	*Only respondents currently active in teaching*
Why do you teach?				ns	None
Already part of the job	19 (79 %)	3 (43 %)	33 (83 %)		
Added by the manager	1 (4 %)	1 (14 %)	1 (3 %)		
Added by yourself	3 (13 %)	2 (29 %)	5 (13 %)		
Added by someone else	1 (4 %)	1 (14 %)	1 (3 %)		
*Enthusiasm about teaching*	*n = 46*	*n = 11*	*n = 63*	*–*	*Only respondents active in teaching since graduation*
Appreciation of teaching task				1 + 2 vs 3, 1 vs 2 + 3, 1 vs 3*	2/0/4
(Very) negative	0 (0 %)	0 (0 %)	0 (0 %)		
Moderate	0 (0 %)	0 (0 %)	4 (7 %)		
(Very) positive	44 (100 %)	11 (100 %)	55 (93 %)		
Preparedness for teaching				1 + 2 vs 3**, 1 vs 2 vs 3*, 1 vs 2 + 3**, 1 vs 3**	2/0/4
(Very) insufficient	0 (0 %)	0 (0 %)	11 (19 %)		
Moderate	5 (11 %)	3 (27 %)	13 (22 %)		
(Very) good	39 (89 %)	8 (73 %)	35 (59 %)		

*BKO* basic university teaching certificate, *SKO* senior university teaching certificate; Values are medians with interquartile range (IQR) or numbers with percentages (%), *ns* not significant

*p < 0.05; **p < 0.01; ***p < 0.001
